# Cloning and Sequencing of Protein Kinase cDNA from Harbor Seal (*Phoca vitulina*) Lymphocytes

**DOI:** 10.1080/10446670410001722195

**Published:** 2004-06

**Authors:** Jennifer C. C. Neale, Thomas P. Kenny, M. Eric Gershwin

**Affiliations:** 1 Department of Internal Medicine, Division of Allergy, Rheumatology, and Immunology University of California Davis CA USA

## Abstract

Protein kinases (PKs) play critical roles in signal transduction and activation of lymphocytes. The identification of PK genes provides a tool for understanding mechanisms of immunotoxic xenobiotics. As part of a larger study investigating persistent organic pollutants in the harbor seal and their possible immunomodulatory actions, we sequenced harbor seal cDNA fragments encoding PKs. The procedure, using degenerate primers based on conserved motifs of human protein tyrosine kinases (PTKs), successfully amplified nine phocid PK gene fragments with high homology to human and rodent orthologs. We identified eight PTKs and one dual (serine/threonine and tyrosine) kinase. Among these were several PKs important in early signaling events through the B- and T-cell receptors (FYN, LYN, ITK and SYK) and a MAP kinase involved in downstream signal transduction. V-FGR, RET and DDR2 were also expressed. Sequential activation of protein kinases ultimately induces gene transcription leading to the proliferation and differentiation of lymphocytes critical to adaptive immunity. PKs are potential targets of bioactive xenobiotics, including persistent organic pollutants of the marine environment; characterization of these molecules in the harbor seal provides a foundation for further research illuminating mechanisms of action of contaminants speculated to contribute to large-scale die-offs of marine mammals via immunosuppression.


*Clinical & Developmental Immunology*, June 2004, Vol. 11 (2), pp. 157–163

Taylor & Francis healthsciences

### Cloning and Sequencing of Protein Kinase cDNA from Harbor Seal (*Phoca vitulina*) Lymphocytes

JENNIFER C.C. NEALE*, THOMAS P. KENNY and M. ERIC GERSHWIN

*Department of Internal Medicine, Division of Allergy, Rheumatology, and Immunology, University of California, One Shields Avenue, Davis, CA 95616, USA*

Protein kinases (PKs) play critical roles in signal transduction and activation of lymphocytes. The identification of PK genes provides a tool for understanding mechanisms of immunotoxic xenobiotics. As part of a larger study investigating persistent organic pollutants in the harbor seal and their possible immunomodulatory actions, we sequenced harbor seal cDNA fragments encoding PKs. The procedure, using degenerate primers based on conserved motifs of human protein tyrosine kinases (PTKs), successfully amplified nine phocid PK gene fragments with high homology to human and rodent orthologs. We identified eight PTKs and one dual (serine/threonine and tyrosine) kinase. Among these were several PKs important in early signaling events through the B- and T-cell receptors (FYN, LYN, ITK and SYK) and a MAP kinase involved in downstream signal transduction. V-FGR, RET and DDR2 were also expressed. Sequential activation of protein kinases ultimately induces gene transcription leading to the proliferation and differentiation of lymphocytes critical to adaptive immunity. PKs are potential targets of bioactive xenobiotics, including persistent organic pollutants of the marine environment; characterization of these molecules in the harbor seal provides a foundation for further research illuminating mechanisms of action of contaminants speculated to contribute to large-scale die-offs of marine mammals via immunosuppression.

*Keywords*: Harbor seal; *Phoca vitulina*; Protein (tyrosine) kinase; Lymphocyte activation and differentiation

### INTRODUCTION

Protein kinases (PKs) play critical roles in cellular functions including signal transduction, cell cycle regulation, cell division and cell differentiation (Hunter *et al.*, 1985; Edelman *et al.*, 1987). Signal transduction controls many critical and complex cellular functions, including activation of lymphocytes in the adaptive immune response. Signal transduction through the B- and T-cell receptors (BCR and TCR, respectively) and cytokine receptors on the surface of lymphocytes occurs largely via tyrosine phosphorylation of intracellular substrates by protein tyrosine kinases (PTKs). Tyrosine kinases (TKs) play a major role in many disorders of cell proliferation, differentiation, survival and migration, which are fundamental to many diseases and abnormalities.

Although research in this area is limited and relatively recent, there is already experimental evidence that certain halogenated aromatic hydrocarbons [HAHs such as polychlorinated biphenyls (PCBs) and 2,3,7,8-tetrachlorodibenzo-*p*-dioxin (TCDD)] and polycyclic aromatic hydrocarbons (PAHs)—affect tyrosine kinases. For example, *in vitro* exposure of murine B lymphocytes to TCDD increased membrane protein phosphorylation and, in particular, stimulated tyrosine-specific protein phosphorylation (Clark *et al.*, 1991). Similarly, certain PAHs have been shown to activate PTKs in human cell lines—specifically, FYN and LCK in T-cells and LYN and SYK in B cells (Archuleta *et al.*, 1993; Mounho and Burchiel, 1998). These authors also reported that mobilization of intracellular calcium was coupled to increased tyrosine phosphorylation, and suggested that PAH-induced PTK activation and increased cellular $Ca^{2+}$ may alter antigen receptor signaling in human B cells.

Disruption of immune function associated with tissue accumulation of halogenated aromatic hydrocarbons in the marine environment has been suggested as a factor altering health in several marine mammal species (Addison, 1989; Tanabe *et al.*, 1994). For example, it has been speculated that contaminant-induced immunosuppression may have contributed to the high mortality observed in several marine mammal populations during recent morbillivirus epizootics (Hall *et al.*, 1992; Aguilar and Borrell, 1994). HAHs and PAHs are known to elicit a broad spectrum of immunotoxic effects in laboratory

*Corresponding author. Tel.: +1-530-752-3286. E-mail: jcneale@ucdavis.edu

ISSN 1740-2522 print/ISSN 1740-2530 online © 2004 Taylor & Francis Ltd
DOI: 10.1080/10446670410001722195

animals (Kerkvliet and Burleson, 1994; White *et al.*, 1994). In marine mammals, PAHs and organochlorines (such as PCBs and DDT) also have been associated with impaired immunological function (Martineau *et al.*, 1994; Lahvis *et al.*, 1995; Ross *et al.*, 1996; Beckmen *et al.*, 2003). Recently, certain PCB and PAH compounds were shown to suppress harbor seal T-cell mitogenesis *in vitro* (Neale *et al.*, 2002).

PKs provide the machinery for the differentiation and activation of lymphocytes, processes critical to cell-mediated immunity and host resistance to pathogens. As part of a larger investigation of potential contaminant-induced immune alterations in the harbor seal, we wished to identify key signal transduction molecules in seal lymphocytes homologous to human and rodent PKs, for use as molecular biomarkers of contaminant-induced changes in kinase gene expression. We describe here the first nucleotide (partial) sequences of protein kinase genes in the harbor seal.

### MATERIALS AND METHODS

### Sample Collection

Free-ranging harbor seals were captured as described previously (Neale *et al.*, 2002) and blood drawn from the extradural vein into sterile evacuated blood collection tubes containing acid citrate dextrose (Becton Dickinson Vacutainer Systems, Franklin Lakes, NJ, USA; NMFS Scientific Research Permit Nos. 555–1565, 373–1575). Material used for this study was collected from three clinically healthy seals caught in central California, including an adult female from San Francisco Bay on 7/16/01, a yearling male from San Francisco Bay on 1/24/02, and a male subadult from Monterey Bay on 2/7/02.

### RNA Isolation

Within 12 h of collection, PBMC were isolated from approximately 8.5 ml whole blood by centrifugation of blood for 20 min at 300*g*. Buffy coats were diluted 1:2 in RPMI 1640 medium (Sigma-Aldrich, St Louis, MO, USA). Mononuclear cells were further purified by density gradient centrifugation (30 min at 700*g*) using 5 ml Histopaque-1077 (Sigma-Aldrich). The resulting white blood cell layer was removed and washed twice in Hank's Balanced Salt Solution (JRH Biosciences, Lenexa, KS, USA) with 10 min centrifugations at 200*g*. The white blood cell pellet was transferred to a 15-ml conical tube and TRIzol reagent (GibcoBrl/Life Technologies, Gaithersburg, MD, USA) was added in increments of 200 μl, with repetitive pipetting to ensure complete lysis of cells, for a total of 1 ml. After 10 min incubation, the mixture was transferred to a 1-ml microcentrifuge tube. RNA was isolated (phenol–chloroform separation) and precipitated from the aqueous phase with isopropanol, according to the manufacturer's instructions. The RNA pellet (approximately 20 ug) was then redissolved in 20 μl diethyl-pyrocarbonate (DEPC)-treated RNase-free water (hereafter, "water") and incubated at 60° C for 10 min.

### RT-PCR

For reverse transcription, approximately 5 ug RNA (in 5 μl water) was added to a mixture of 1 μl (0.5 ug) oligo(dT) primers, 1 μl dNTP Mix (10 mM), and 5 μl water. This mixture was incubated at 70°C for 10 min then cooled on ice. Next, 4 μl of 5 × First Strand Buffer, 2 μl of DTT (0.1 M) and 1 μl (10 units) RNase inhibitor were added to the first mixture and incubated at 42°C for 2 min. Lastly, 1 μl (200 units) Superscript II reverse transcriptase was added for a total volume of 20 μl. This was incubated for 42°C for 50 min. The reverse transcriptase enzyme was heat-killed at 70°C for 15 min. All reagents supplied from Invitrogen (Carlsbad, CA, USA).

Various protein kinase transcripts were PCR-amplified using degenerate primers derived from the conserved motifs DFG (5′) and DVW (3′) within the catalytic domains of (human) PTK. These have been described in detail previously (Robinson *et al.*, 1996; Kung *et al.*, 1998; Lin *et al.*, 1998). Briefly, DFG is present in virtually all kinases, whereas DVW is primarily encoded by tyrosine kinases, although a small number of serine kinases can also be primed. These two motifs bound a conserved area of approx. 50 aa which, in phosphorylated tyrosine kinases, interact only with target molecules that contain SH2 domains. The 5′ primer used to encode the amino acid sequence K[V/I][S/C/G]DFG is represented by: 5′-AAR RTT DCN GAY TTY GG. The 3′ primer used to encode the amino acid sequence DVW[S/A][F/Y] is represented by: 5′-RHA IGM CCA IAC RTC. The mixed bases were defined as follows: $N = A/C/T/G$, $D = A/T/G$, $H = A/T/C$, $R = A/G$, $Y = C/T$, $M = A/C$ and I = deoxyinosine.

PCR reactions (25 μl) contained 1 μl cDNA, 2.5 μl 10 × buffer, 0.5 μl each of 3′, 5′, and dNTP Mix (10 mM), 1.5 μl $MgCl_2$ (25 mM), 0.5 μl (2.5 units) *Taq* polymerase, and 18 μl water (PCR profile: 94°C, 11 min; 52°C, 1 min; 72°C, 1.5 min; 31 cycles; 72°C, 10 min). PCR products were electrophoresced in an agarose gel. Due to the roughly even spacing between DFG and DVW in all kinases, we expected and obtained a relatively homogeneous PCR product of ~170 bp. This band was excised and DNA purified using the QIAEX II gel extraction kit (Qiagen, Valencia, CA, USA).

### Cloning, Transformation, and Sequencing

Purified fragments were cloned into a plasmid vector using the TOPO TA Cloning Kit for Sequencing (Version H, Invitrogen) and the resulting recombinants were used to electro-transform *E. coli*, according to the manufacturer's instructions. Plasmid DNA was isolated using the Qiagen Plasmid Mini Kit (Qiagen, Valencia, CA, USA). PCR and

FIGURE 1 Nucleotide sequence alignment of harbor seal PK cDNA. DFG (5′) and DVW (3′) domains are bolded and underlined. Sequences have been submitted to Genbank with the following accession numbers: LYN+, #AY611615; LYN, #AY611614; FYN, #AY611616; V-FGR, #AY611622; ITK, #AY611617; SYK, #AY611618; DDR2, #AY611620; MAPKK3, #AY611619; RET, #AY611621.

FIGURE 2 Deduced amino acid sequences corresponding to nucleotide sequences of nine harbor seal PK genes. DFG and DVW motifs underlined. Reading frame starting base and total length (amino acids) given.

TABLE I Homology (% identities) between harbor seal PKs and human, rat (*Rattus norvegicus*) and mouse (*Mus musculus*) orthologs based on sequences between the DFG and DVW domains (non-inclusive)

| | Nucleotide | | | | Amino acid | | | |
|---|---|---|---|---|---|---|---|---|
| Gene | Human | Rat | Mouse | # Bases | Human | Rat | Mouse | # Amino acids |
| FYN | 91 | 88 | 90 | 111 | 100 | 100 | 100 | 37 |
| LYN | 93 | 92 | 89 | 111 | 100 | 100 | 100 | 37 |
| ITK | 92 | 92 | 89 | 111 | 100 | 100 | 100 | 37 |
| SYK | 93 | 92 | 93 | 117 | 100 | 97 | 100 | 39 |
| MAPKK3 | 91 | 87 | 87 | 117 | 97 | 97 | 97 | 39 |
| DDR2 | 95 | 96 | 94 | 114 | 100 | 100 | 100 | 38 |
| RET | 95 | 90 | 88 | 114 | 100 | 100 | 100 | 38 |
| v-FGR | 89 | 95 | 89 | 111 | 92 | 92 | 92 | 37 |

gel electrophoresis were used at several steps to screen and select positive clones. In addition, because half (8/16) of the clones initially analyzed were identified as ITK, colonies were also screened using restriction endonuclease digestion, in this way we were able to selectively analyze non-ITK clones. Diversity of selection was further enhanced by visualization of slight size variations among positive clones using acrylamide gel electrophoresis. Inserts were sequenced at the DNA sequencing facility (University of California, Davis) using the M13 Reverse universal primer. We used the BLAST program (Altschul *et al.*, 1997) to search protein and DNA databases for sequence similarities.

### RESULTS

Clones positive for PK transcripts were generated from all three seals, and multiple clones were identified for most kinases. In all cases, replicate sequences were identical between the DFG and DVW domains. Because the greatest volume of blood (and thus lymphocyte RNA) was obtained from the yearling male, the majority (and greatest diversity) of PK sequences came from this individual.

We obtained nucleotide sequences encoding nine distinct protein kinases, including eight PTK—FYN, LYN and LYN+, ITK, SYK, v-FGR, DDR2 and RET—and one dual kinase (i.e. having both tyrosine and serine/threonine kinase activities), MAPKK3 (Fig. 1). LYN+, a variant of LYN tyrosine kinase, contained an insert of 29 bases beginning at 72, but was otherwise identical in nucleotide sequence to LYN. All other sequences were of the expected length (165–171 nucleotides).

Deduced protein sequences corresponding to the nine harbor seal PK nucleotide sequences are presented in Fig. 2. The 29-base insert of Lyn+ causes a shift in reading frame; consequently, the DVW motif is not encoded, but an open reading frame is maintained.

Identification was based on homology with published cDNA sequences. Nucleotide and deduced amino acid sequences were highly similar to human and rodent orthologs (Table I). Additional information based on the corresponding human proteins is provided in Table II.

### DISCUSSION

Here, we demonstrated the ability to prime phocid PK cDNA using a human-based degenerate PCR primer mix and we identified, for the first time, protein kinases in an organism within the order Carnivora. Among the kinases identified were several key players involved in signal transduction through the BCR/TCR and activation of B- and T-cells— namely, FYN, LYN, SYK and MAPKK3.

The Src-family kinases FYN and LYN (together with BLK in B cells and LCK in T cells) are responsible for early events in BCR and TCR signaling (Qian and Weiss, 1997; Tsubata and Wienands, 2001). BCR proximal signaling occurs within “lipid rafts” and depends on the tyrosine kinase activity of LYN

TABLE II Family membership, synonyms and human (*Hs*) protein sequence length and molecular weight, for PKs identified in harbor seal

| Gene | Family | Synonyms | *Hs* seq. length (aa) | *Hs* MW (kDa) |
|---|---|---|---|---|
| FYN | SRC-A | SLK, SYN | 536 | 60.62 |
| LYN | SRC-B | | 511 | 58.44 |
| ITK | TEC | TSK, EMT, PSCTK2 | 620 | 71.83 |
| SYK | SYK | Spleen tyrosine kinase | 635 | 72.07 |
| MAPKK3 | SER/THR | MAP2K3, MKK3, MEK3 | 318 | 37.17 |
| v-FGR | SRC | GR-FeSV oncogene | 529 | 59.46 |
| DDR2 | DDR | TKT, TYRO10, NTRKR3 | 913 | 101.09 |
| RET | RET | MEN2A/B, HSCR1, MTC1 | 1072 | 119.82 |

TABLE III Predicted fragment lengths from nine harbor seal PK genes following restriction enzyme digestion with 13 endonucleases

| Enzyme | LYN+ | LYN | FYN | v-FGR | ITK | SYK | DDR2 | MAPKK3 | RET |
|---|---|---|---|---|---|---|---|---|---|
| BglI | | | | 104 | | | | | |
| MscI | | | 32 | | | | | | |
| HphI | | | | | 97 | 103 | 62 | 25 | |
| ApoI | 106 | 77 | | | | | 125 | | |
| BbvI | | | 119 | 94 | 141 | | | 62 | |
| AciI | | | | | 121 | 159 | 91 | | |
| BceAI | | | 111 | | | | | | |
| SfaNI | | | | | 104 | 123 | 120 | | |
| BsaAI | | | | | | | | | 60 |
| BsmAI | 87 | | | 41 | | | | | 113 |
| AflIII | | | | | 141 | | | | 143 |
| DdeI | 72 | | | | | | | | |
| BpmI | 113 | 84 | | | | 118,90 | | | |

Data generated in NEBcutter (version 2.0, New England Biolabs, Inc., http://tools.neb.com/NEBcutter2).

(Tsubata and Wienands, 2001; Chakravarty *et al.*, 2002). Likewise, FYN is critical for initiating TCR signaling and plays an important role in T-cell development as well (Howe and Weiss, 1995). Specifically, phosphorylation of the tyrosines in immunoreceptor tyrosine-based activation motifs (ITAMs) by Src-family PTKs serves as the initial intracellular signal indicating that the lymphocyte has detected its specific antigen (Janeway *et al.*, 1999).

SYK is a critical PTK in B-cell antigen receptor signaling and B-cell development (Cheng *et al.*, 1995; Turner *et al.*, 1995). Once the ITAMs in the receptor cytoplasmic tails have been phosphorylated, ITAMs in B cells bind SYK (ZAP-70 in T cells). Until SYK has been bound, it is inactive enzymatically. To be activated, it also must be phosphorylated; for this reason, SYK is thought to be important in the negative regulation of receptor TK-coupled signaling processes as well. Once activated, SYK phosphorylates target proteins to initiate a cascade of intracellular signaling molecules (Janeway *et al.*, 1999).

MAPKK3 (mitogen-activated protein kinase kinase 3) is integral to the MAP kinase signaling cascade, one of several pathways leading to activation of transcription factors in the nucleus. The MAP kinase pathway is of great importance in both BCR/TCR signaling and T-cell development (Alberola-Ila *et al.*, 1995; Li *et al.*, 1996; Abbas and Lichtman, 2003). Activated kinase SYK (or ZAP-70 in T cells) activates phopholipase C-$\gamma$ (leading to the diacylglycerol and inositol trisphosphate pathways) and guanine-nucleotide exchange factors (GEFs). GEFs activate small G proteins, which in turn activate MAP kinase cascades. The role of the dual kinase MAPKK3 is to activate, via phosphorylation of a threonine and a tyrosine residue, particular MAP kinases, which then activate transcription factors to induce specific gene transcription leading to cell proliferation and differentiation.

ITK (murine IL-2 inducible T-cell kinase) is expressed in T lymphocytes (and NK cells) and is required for normal T-cell development (Janeway *et al.*, 1999). There is strong evidence that ITK regulates TCR signaling, but the mechanism underlying this role has not been determined (Liao and Littman, 1995). The Tec family PTK preferentially expressed in T cells is ITK (Qian and Weiss, 1997). ITK was disproportionately represented (50%) in our clones, suggesting that ITK may be highly expressed in harbor seal lymphocytes, although the small scale of this preliminary study prevents quantitative assessment of gene expression.

The relevance to signal transduction and lymphocyte activation of the lesser-known TKs DDR2, v-FGR and RET is not clear, although all three appear to be involved in various disease processes. The recently identified receptor tyrosine kinase DDR2 (discoidin domain receptor family, member 2) is unusual in that it is activated by fibrillar collagens (types I and III) rather than a growth factor-like peptide (Vogel *et al.*, 1997; Leitinger, 2003). In addition to its role as a DDR2 ligand, fibrillar collagen matrix can sequester and provide binding sites for immune mediators such as cytokines and chemokines which may lead to local tissue damage (Somasundaram *et al.*, 2002). The activation of DDR2 also mediates the over-expression of matrix metalloproteinase 1 in cells, which is thought to be involved in the metastasis of some tumors (Wang *et al.*, 2001).

V-FGR and RET were identifed as (proto-) oncogenes. V-FGR (feline sarcoma viral oncogene for fibroblast growth factor) arises from a recombination of a cellular structural gene (gamma actin) with a tyrosine kinase gene (*c-fgr*) (Baker *et al.*, 1998). The transforming activity of v-FGR appears to lie in its TK, which may activate substrates not normally exposed to tyrosine phosphorylation (Sugita *et al.*, 1989). Certain gene rearrangements of the RET receptor TK kinase proto-oncogene are responsible for cancer pathogenesis (Bongarzone *et al.*, 2003). Activating mutations lead to the expression of deregulated products characterized by ligand-independent activation of the intrinsic tyrosine kinase of RET (Lanzi *et al.*, 2003).

Because of their critical role in adaptive immunity, protein kinases (especially PTK) may represent important targets of immunomodulation by xenobiotics in marine mammals. Harbor seal sequences for PTK could serve as molecular biomarkers in semi-quantitative profiling of kinase gene expression. For example, differential expression of specific PTK, following *in vitro* exposures of seal lymphocytes to model compounds, could be identified via RT-PCR and restriction enzyme digest analysis. This technique has been applied previously to human samples to characterize kinase expression in disease (Robinson *et al.*, 1996; Kung *et al.*, 1998; Mao *et al.*, 2002). In this approach, subsamples of labeled DNA (i.e. radioactive or fluorescent tags) are digested with multiple restriction enzymes, after which digested products are resolved via acrylamide gel electrophoresis. Nucleotide sequences of PK and known restriction sites of endonucleases are utilized to identify kinases and assess differential expression, e.g. for treated vs. control samples. Table III shows an optimized digest for the nine harbor seal PK presented here.

### *Acknowledgements*

We thank the individuals and organizations responsible for harbor seal blood sampling, including J. Harvey and S. Oates (Moss Landing Marine Laboratories, Moss Landing, CA), S. Allen, E. Grigg, and D. Green (Richmond Bridge Harbor Seal Survey/San Francisco State Univ., San Francisco, CA), and volunteers from these groups as well as The Marine Mammal Center (Sausalito, CA). R. Tjeerdema, D. Anderson, J. Harvey, and B. Sacks provided helpful reviews of the manuscript. This project was supported in part by the California Department of Fish and Game's Oil Spill Response Trust Fund through the Oiled Wildlife Care Network at the Wildlife Health Center, School of Veterinary Medicine, University of California, Davis. Additional funding was provided to JN by grants from the UC Marine Council (#02 T CEQI 03 0104) and the NIH (#5 T32 ES07059-25 Traineeship in Environmental Toxicology).

### *References*

Abbas, A.K. and Lichtman, A.H. (2003) Cellular and Molecular Immunology, 5th Ed. (Saunders, Philadelphia, PA, USA).

Addison, R.F. (1989) "Organochlorines and marine mammal reproduction", *Can. J. Fish Aquat. Sci.* **46**, 360–368.

Aguilar, A. and Borrell, A. (1994) "Abnormally high polychlorinated biphenyl levels in striped dolphins (*Stenella coeruleoalba*) affected by the 1990–92 mediterranean epizootic", *Sci. Tot. Environ.* **154**, 237–247.

Alberola-Ila, J., Forbush, K.A., Seger, R., Krebs, E.G. and Permulter, R.M. (1995) "Selective requirement for MAP kinase activation in thymocyte differentiation", *Nature* **373**, 620–623.

Altschul, S.F., Madden, T.L., Schaffer, A.A., *et al.* (1997) "Gapped BLAST and PSI-BLAST: a new generation of protein database search programs", *Nucleic Acids Res.* **25**, 3389–3402.

Archuleta, M.M., Schieven, G.L., Ledbetter, J.A., Deanin, G.G. and Burchiel, S.W. (1993) "7,12-Dimethylbenz[a]anthracene activates protein-tyrosine kinases Fyn and Lck in the HPB-ALL human T-cell line and increases tyrosine phosphorylation of phospholipase C-γ1, formation of inositol 1,4,5-triphosphate, and mobilization of intracellular calcium", *Proc. Natl Acad. Sci.* **90**, 6105–6109.

Baker, S.J., Cosenza, S.C. and Reddy, E.P. (1998) "The role of v-FGR myristoylation and the Gag domain in membrane binding and cellular transformation", *J. Virol.* **249**, 1–11.

Beckmen, K.B., Blake, J.E., Ylitalo, G.M., Stott, J.L. and O'Hara, T.M. (2003) "Organochlorine contaminant exposure and associations with hematological and humoral immune functional assays with damage as a factor in free-ranging northern fur seal pups (*Callorhinus ursinus*)", *Mar. Pollut. Bull.* **46**, 594–606.

Bongarzone, I., Carniti, C., Perego, C., Mondellini, P. and Pierotti, M.A. (2003) "RETMEN2A and RETMEN2B oncoproteins are targets of PP1 inhibitor", *Tumori* **89**, 550–552.

Chakravarty, L., Zabel, M.D., Weis, J.J. and Weis, J.H. (2002) "Depletion of Lyn kinase from the BCR complex and inhibition of B cell activation by excess CD21 ligation", *Int. Immunol.* **14**, 139–146.

Cheng, A.M., Rowley, B., Pao, W., Hayday, A., Bolen, J.B. and Pawson, T. (1995) "Syk tyrosine kinase required for mouse viability and B-cell development", *Nature* **378**, 303–306.

Clark, G.C., Blank, J.A., Germolec, D.R. and Luster, M.I. (1991) "2,3,7,8-Tetrachlorodibenzo-p-dioxin stimulation of tyrosine phosphorylation in B lymphocytes: potential role in immunosuppression", *Mol. Pharmacol.* **39**, 495–501.

Edelman, A.M., Blumenthal, D.K. and Krebs, E.G. (1987) "Protein serine/threonine kinases", *Annu. Rev. Biochem.* **56**, 567–613.

Hall, A.J., Law, R.J., Harwood, J., *et al.* (1992) "Organochlorine levels in common seals (*Phoca vitulina*) which were victims and survivors of the 1988 phocine distemper epizootic", *Sci. Tot. Environ.* **115**, 145–162.

Howe, L.R. and Weiss, A. (1995) "Multiple kinases mediate T-cell-receptor signaling", *Trends Biochem. Sci.* **20**, 59–64.

Hunter, T. and Cooper, J.A. (1985) "Protein-tyrosine kinases", *Annu. Rev. Biochem.* **54**, 897–930.

Janeway, C.A., Travers, P., Walport, M. and Capra, J.D. (1999) Immunobiology: The Immune System in Health and Disease, 4th Ed. (Garland Publishing, New York, NY, USA).

Kerkvliet, N.I. and Burleson, G.R. (1994) "Immunotoxicity of TCDD and related halogenated aromatic hydrocarbons", In: Dean, J.H., Luster, M.I., Munson, A.E. and Kimber, I., eds, Immunotoxicology and Immunopharmacology, 2nd Ed. (Raven Press, New York, NY, USA), pp 97–121.

Kung, H-J., Chen, H-C. and Robinson, D. (1998) "Molecular profiling of tyrosine kinases in normal and cancer cells", *J. Biomed. Sci.* **5**, 74–78.

Lahvis, G.P., Wells, R.S., Kuehl, D.W., Stewart, J.L., Rhinehart, H.L. and Via, C.S. (1995) "Decreased lymphocyte responses in free-ranging bottlenose dolphins (*Tursiops truncatus*) are associated with increased concentrations of PCBs and DDT in peripheral blood", *Environ. Health Perspect.* **103**(Suppl. 4), 67–72.

Lanzi, C., Cassinelli, G., Cuccuru, G., Zanchi, C., Laccabue, D. and Zunino, F. (2003) "RET/PTC oncoproteins: molecular targets of new drugs", *Tumori* **89**, 520–522.

Leitinger, B. (2003) "Molecular analysis of collagen binding by the human discoidin domain receptors, DDR1 and DDR2. Identification of collagen binding sites in DDR2", *J. Biol. Chem.* **278**, 16761–16769.

Li, W., Whaley, C.D., Mondino, A. and Mueller, D.L. (1996) "Blocked signal transduction to the ERK and JNK protein kinases in anergic CD4+ T cells", *Science* **271**, 1272–1276.

Liao, X.C. and Littman, D.R. (1995) "Altered T cell receptor signaling and disrupted T cell development in mice lacking Itk", *Immunity* **3**, 757–769.

Lin, J-S., Lu, C-W., Huang, C-J., *et al.* (1998) "Protein-tyrosine kinase and protein-serine/threonine kinase expression in human gastric cancer cell lines", *J. Biomed. Sci.* **5**, 101–110.

Mao, T.K., Yasunori, K., Kenny, T.P., *et al.* (2002) "Elevated expression of tyrosine kinase DDR2 in primary biliary cirrhosis", *Autoimmunity* **35**, 521–529.

Martineau, D., De Guise, S., Fournier, M., *et al.* (1994) "Pathology and toxicology of beluga whales from the St. Lawrence Estuary, Quebec, Canada. Past, present, and future", *Sci. Tot. Environ.* **154**, 201–215.

Mounho, B.J. and Burchiel, S.W. (1998) "Alterations in human B cell calcium homeostasis by polycyclic aromatic hydrocarbons: possible associations with cytochrome p450 metabolism and increased protein tyrosine phosphorylation", *Toxicol. Appl. Pharmacol.* **149**, 80–89.

Neale, J.C.C., Van de Water, J.A., Harvey, J.T., Tjeerdema, R.S. and Gershwin, M.E. (2002) "Proliferative responses of harbor seal (*Phoca vitulina*) T lymphocytes to model marine pollutants", *Dev. Immunol.* **9**, 215–221.

Qian, D. and Weiss, A.T. (1997) "Cell antigen receptor signal transduction", *Curr. Opin. Cell Biol.* **9**, 205–212.

Robinson, D., He, F., Pretlow, T. and Kung, H-J. (1996) "A tyrosine kinase profile of prostrate carcinoma", *Proc. Natl Acad. Sci.* **93**, 5958–5962.

Ross, P.S., De Swart, R.L., Addison, R.F., van Loveren, H., Vos, J.G. and Osterhaus, A.D.M.E. (1996) "Contaminant-induced immunotoxicity in harbour seals: wildlife at risk?", *Toxicol* **112**, 157–169.

Somasundaram, R., Ruehl, M., Schaefer, B., *et al.* (2002) "Interstitial collagens I, III, and VI sequester and modulate the multifunctional cytokine oncostatin M", *J. Biol. Chem.* **277**, 3242–3246.

Sugita, K., Gutkind, J.S., Katamine, S., Kawakami, T. and Robbins, K.C. (1989) "The actin domain of Gardner-Rasheed feline sarcoma virus inhibits kinase and transforming activities", *J. Virol.* **63**, 1715–1720.

Tanabe, S., Iwata, H. and Tatsukawa, R. (1994) "Global contamination by persistent organochlorines and their ecotoxicological impact on marine mammals", *Sci. Tot. Environ.* **154**, 163–177.

Tsubata, T. and Wienands, J. (2001) "B cell signaling. Introduction", *Int. Rev. Immunol.* **20**, 675–678.

Turner, M., Mee, P.J., Costello, P.S., *et al.* (1995) "Perinatal lethality and blocked B-cell development in mice lacking the tyrosine kinase Syk", *Nature* **378**, 298–302.

Vogel, W., Gish, G.D., Alves, F. and Pawson, T. (1997) "The discoidin domain receptor tyrosine kinases are activated by collagen", *Mol. Cell* **1**, 13–23.

Wang, J.C., Liu, X.P., Nie, X.Y., *et al.* (2001) "Expression, purification, and functional identification of extracellular part of discoidin domain receptor 2", *Sheng Wu Hua Xue Yu Sheng Wu Wu Li Xue Bao (Shanghai)* **33**, 647–652.

White, K.L., Jr., Kawabata, T.T. and Ladics, G.S. (1994) "Mechanisms of polycyclic aromatic hydrocarbon immunotoxicity", In: J.H. Dean, M.I. Luster, A.E. Munson and I. Kimber, eds, Immunotoxicology and Immunopharmacology, 2nd Ed. (Raven Press, New York, NY, USA), pp 123–142.

